# Improvement of Apraxia With Augmented Reality: Influencing Pantomime of Tool Use via Holographic Cues

**DOI:** 10.3389/fneur.2021.711900

**Published:** 2021-08-26

**Authors:** Nina Rohrbach, Carmen Krewer, Lisa Löhnert, Annika Thierfelder, Jennifer Randerath, Klaus Jahn, Joachim Hermsdörfer

**Affiliations:** ^1^Technical University Munich, Chair of Human Movement Science, Munich, Germany; ^2^Schön Klinik Bad Aibling, Bad Aibling, Germany; ^3^Lurija Institute for Rehabilitation Sciences and Health Research at the University of Konstanz, Konstanz, Germany; ^4^Ludwig-Maximilians University of Munich, University Hospital Grosshadern, Munich, Germany

**Keywords:** virtual reality, apraxia, pantomime of tool use, stroke, hologram, sense of presence, visual cues

## Abstract

**Background:** Defective pantomime of tool use is a hall mark of limb apraxia. Contextual information has been demonstrated to improve tool use performance. Further, knowledge about the potential impact of technological aids such as augmented reality for patients with limb apraxia is still scarce.

**Objective:** Since augmented reality offers a new way to provide contextual information, we applied it to pantomime of tool use. We hypothesize that the disturbed movement execution can be mitigated by holographic stimulation. If visual stimuli facilitate the access to the appropriate motor program in patients with apraxia, their performance should improve with increased saliency, i.e., should be better when supported by dynamic and holographic cues vs. static and screen-based cues.

**Methods:** Twenty one stroke patients and 23 healthy control subjects were randomized to mime the use of five objects, presented in two *Environments* (Screen vs. Head Mounted Display, HMD) and two *Modes* (Static vs. Dynamic) resulting in four conditions (Screen^Stat^, Screen^Dyn^, HMD^Stat^, HMD^Dyn^), followed by a real tool demonstration. Pantomiming was analyzed by a scoring system using video recordings. Additionally, the sense of presence was assessed using a questionnaire.

**Results:** Healthy control participants performed close to ceiling and significantly better than patients. Patients achieved significantly higher scores with holographic or dynamic cues. Remarkably, when their performance was supported by animated holographic cues (e.g., striking hammer), it did not differ significantly from real tool demonstration. As the sense of presence increases with animated holograms, so does the pantomiming.

**Conclusion:** Patients' performance improved with visual stimuli of increasing saliency. Future assistive technology could be implemented upon this knowledge and thus, positively impact the rehabilitation process and a patient's autonomy.

## Introduction

Apraxia occurs in 30–50% of patients after left brain damage (LBD) (1, 2) and frequently co-occurs with other syndromes, such as aphasia or neglect (3–6). Limb apraxia refers to a higher-order motor disorder of learned purposive movement skills not caused by deficits of elemental motor or sensory systems (7) that may also affect activities of daily living (ADL) (8, 9). Patients show impairments in planning or producing motor actions. Typically, they have problems with gesture imitation, pantomimed tool use, and actual tool use (4, 10, 11). In the pantomime of tool use task patients are asked to produce an action without holding the object in their hand (12). Pantomiming requires both, motor-cognitive (e.g., the spatial configuration of the body, hands and movements) and communicative processes, including the simulative demonstration and integration of semantic and motor features of the underlying tool use action, requiring a heightened demand on the working memory processes (5, 10, 13, 14). Pantomime of tool use is considered as very sensitive in detecting the presence of limb apraxia; typically the pantomime mode appears more sensitive as compared to actual tool use mode (3, 15), however performance measures across these modes correlate and individual patterns appear stable (16, 17). While both modes may retrieve similar concepts, differences may be represented by missing visuotactile feedback, i.e., the absence of mechanical interaction and cues from real objects, the heightened demand on imagery and the translation from mental images to motor execution (5, 10, 11, 16, 18, 19). Contextual information may provide critical cues facilitating the access to an adequate motor concept and may constrain the possibilities for action production (15–17). While tactile feedback alone, such as a stick that resembles the handle of a tool, seems to be inefficient in evoking the correct motor program of an action (20, 21), several studies underlined the role of visual feedback (11, 17, 22). In this regard, it has been shown that the perception of object affordances (i.e., action possibilities offered by the environment and the object's properties) and its visual attributes is influenced by its visuo-perceptual context, such as thematic and functional properties but also by space (23).

Augmented reality (AR) technology provides a unique way to study the contributions of visual information during pantomiming and may help understand the underlying mechanisms of apraxia. This new technology allows manipulating the experimental setting by providing different contextual information. In contrast to virtual reality, in which the user is often immersed in a completely synthetic environment, in AR the user's real environment is not replaced but rather enriched by spatially aligned virtual objects (24). In mixed reality training scenarios, a higher sense of presence, defined as the psychological product of technological immersion (25), is suggested to enhance motor performance (26–28). AR systems are advantageous over virtual reality in providing a better sense of presence and reality judgments because users can still see their body parts when interacting with virtual objects (29). These virtual objects or holograms, herein referred to as the perception of a computer generated object through stereo imaging, can provide detailed visual contextual information about the properties of the object (e.g., size or structure) and its functioning (e.g., a moving hologram showing its intention) by creating a realistic illusion in three dimensions (30). Practicing in a salient environment by using meaningful and context-specific cues is related to induced plasticity, increased motor learning and a transfer to other tasks (31). Saliency is a strong predictor of attention and gaze allocation and as such a crucial factor in most everyday visual tasks and everyday functioning (32–34). While visual salience refers to objective attributes compared to its surroundings (e.g., object color and structure), semantic salience defines associations with an object (e.g., memories or personal importance) and depends on the user (35). We suggest holograms to function as cues with high visual and semantic salience, which might support motor actions in patients with apraxia. This is in line with the most recent concept of “action reappraisal” by Federico and Brandimonte (23), a reasoning-based approach in human tool-use processing, suggesting that tool use actions utilize multiple sources of information, including affordances and contextual conditions.

The main objective of this study was to test the hypothesis that the disturbed movement execution in stroke patients with apraxia can be mitigated by AR stimulation during pantomime tasks. If visual stimuli facilitate the access to the appropriate motor program in patients with apraxia, the performance should improve with cues of higher saliency and more contextual information. Specifically, we consider dynamic holographic tools presented through a Head Mounted Display (HMD) as stimuli with higher salience because the moving character on the one side and the holographic nature (i.e., three-dimensionality) on the other side should attract more attention than two-dimensional static images of a tool, enhancing the perception of the object in this way (33, 36). The enriched contextual environment (e.g., detailed object features such as structure) and the overall realism that is conveyed by these properties should provide more cognitive cues (37). Further, little is known yet as to the impact of the induced sense of presence in virtual environments on motor performance in stroke rehabilitation (26). We suggested the enriched conditions to evoke higher presence, and expected to observe an association between increased presence and pantomime performance. A better understanding of the technological properties (e.g., visual saliency) and user attributes (e.g., presence) that contribute to motor performances in augmented environments may further inform decisions about their use in overall stroke rehabilitation.

## Methods

### Participants

This study was conducted at the neurorehabilitation hospital Schoen Clinic Bad Aibling (Germany). From April 2019 to December 2019, we included a total of 49 participants (25 patients with LBD and 24 healthy age-matched control persons) who fulfilled the eligibility criteria: (1) stroke in the left hemisphere with signs of apraxia (or no stroke in controls), (2) normal or corrected-to-normal vision, (3) sufficient cognitive ability to understand and follow task instructions (tested prior to the study), (4) no other neurological, psychiatric diseases or poor general condition affecting testing (i.e., the patient had to be able to sit for the duration of the experiment). Healthy control participants were recruited via poster announcements distributed in the clinic and University and self-registration. The sample size was based on an estimate on earlier studies comparing different execution conditions for similar actions, in which significant effects were found in comparable samples (*n* = 23 per group) (15, 17). The study was approved by the Ethics Committee of the Medical Faculty of the Technical University of Munich and all participants or their legal representatives provided written informed consent prior to testing, which was performed in accordance to the declaration of Helsinki. The protocol was prospectively registered with the German Clinical Trials Register (DRKS) on 22 September 2018 (TrialID = DRKS00015464, Universal Trial Number = U1111-1220-6410).

### Trial Design

**Figure 1:**
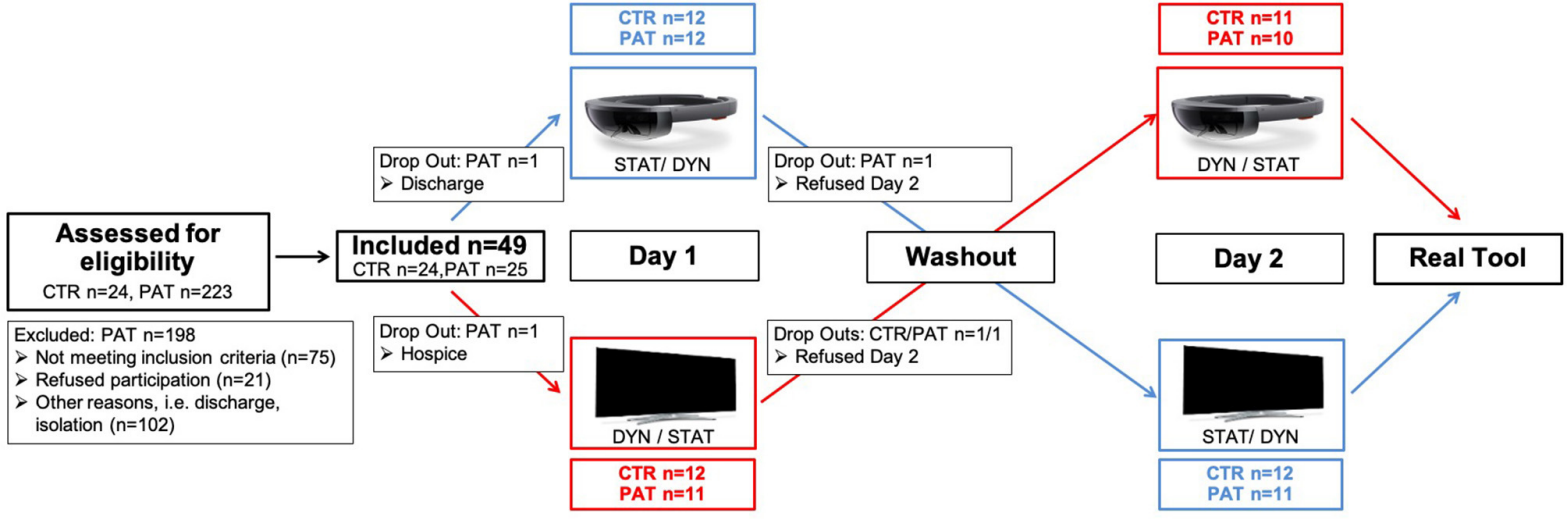
Patients' flow through the study. All participants had to perform the pantomime task twice (Static/Dynamic) in each Environment (HMD/Screen), followed by the real tool condition. The washout time was set to >24 h and did not include any additional tasks. Three participants only completed Day 1 and were excluded from further analyses.

**Figure 2:**
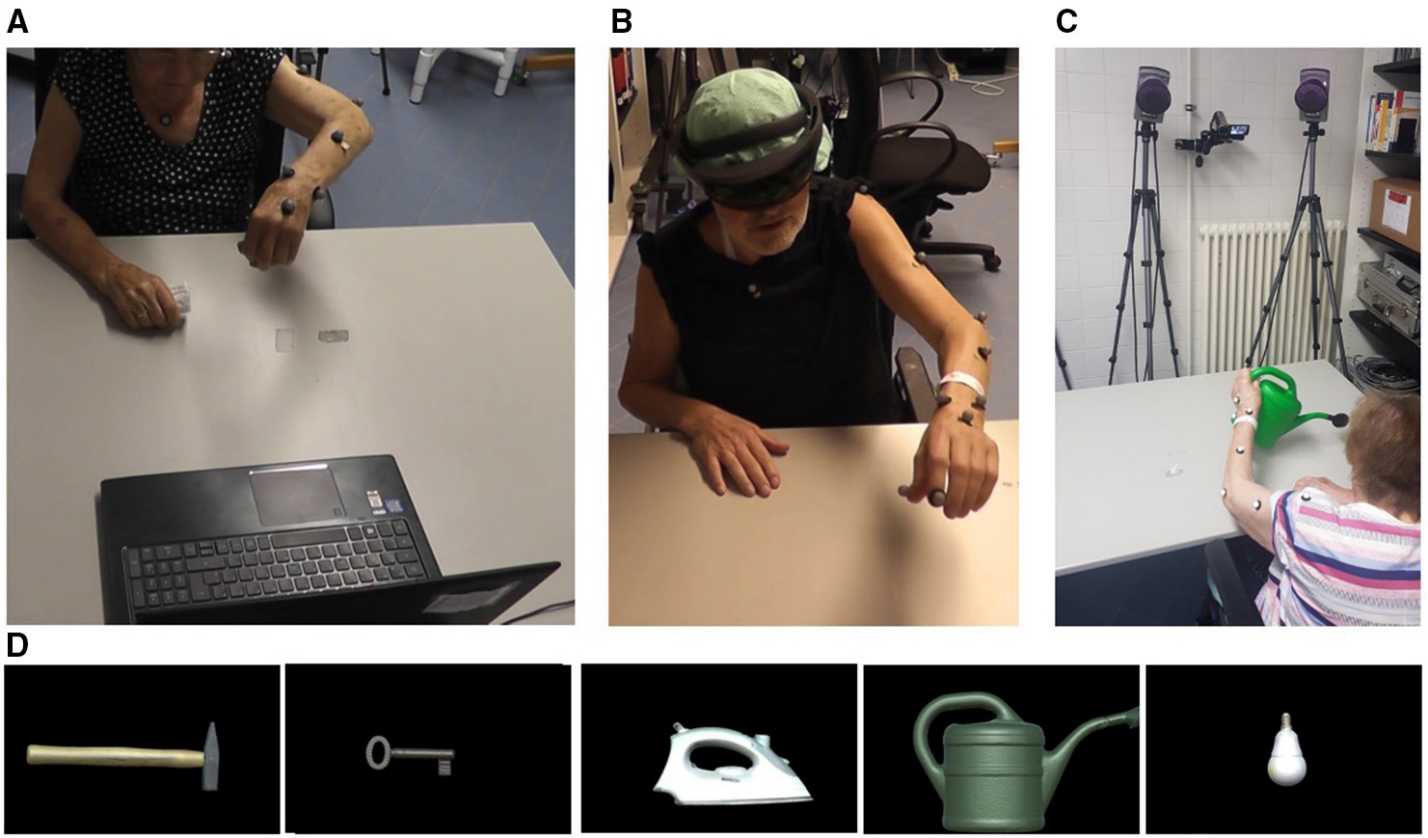
Third person perspective of the experimental setup. **(A)** Screen condition, **(B)** HMD condition, **(C)** Real Tool condition; and **(D)** the first-person perspective of the five objects depicted as screen-based images. Only the tools and not the target items were shown (i.e., the hammer, but not a nail).

Within this randomized crossover study, we tested the influence of varying types of visual stimuli with different degrees of saliency to determine the most effective way of support. Participants had to mime the use of five common objects (hammer, flat-iron, watering can, key, electric bulb) with variable combinations of visual input. On the 1st day, they were randomized 1:1 via sealed envelopes to begin with one of the testing **Environments** (Screen vs. HMD), of which each testing environment was randomized 1:1 to start with one of the testing **Modes** (Static vs. Dynamic). After a 24 h “washout” period, the same task was performed starting with the other testing environment, ending up with four **different combinations**: Screen^Stat^, Screen^Dyn^, HMD^Stat^, HMD^Dyn^ (Figure 1). Each object was presented four times in a row whereas the first presentation was designed as a familiarization where no action was required, to ensure that participants were able to see the images and minimize an influence of visuo-spatial deficits. The order of object presentation was balanced for these four combinations, and held constant for both testing days (i.e., one out of five predefined sequences of object presentations was assigned to each participant). In the screen environment, participants were supported by images of the objects presented on a laptop monitor (15.6-inch, 1,920 x 1,080-pixel resolution), whereby the viewing distance was held constant among all participants (i.e., in a reachable zone of 70 cm when leaning forwards). In the HMD environment, participants wore the Microsoft HoloLens device (1st generation) to view holographic images. In the dynamic mode, one could see the individual tool moving (e.g., striking hammer) while in the static mode the tool remained still (see Supplementary Videos 2, 3). At the end of day 2 after all four conditions were completed, participants had to demonstrate the use of the real tool (in the absence of the target object) that was placed on the table in a standardized way (i.e., the tools were aligned in accordance with the other testing environments, i.e., oriented to promote an action with the left hand as shown in Figure 2D), not accompanied by any additional visual input (“Real Tool” condition).

Participants were seated in front of a table, either facing the screen or wearing the HMD (Figure 2). To familiarize with the HMD a practice holographic object, i.e., a red paper boat (see Supplementary Video 1), was presented accompanied by a standardized explanation of its main technical feature and current limitation of a limited field of view in HoloLens (1st generation). Practice items were included at the beginning of each day by showing printed objects to the participants (fork—corkscrew—saw), and task comprehension was assumed when participants at least attempted to produce a meaningful movement, based on the DILA-S pantomime task recommendations (13). In all conditions participants were verbally instructed by the experimenter (e.g. “please show me how to pound in a nail with a hammer”) as described in (13) and were allowed to start miming as soon as the picture of the object became visible. Their movements were videotaped for later observational evaluation. They used their left hand (non-paretic) in all conditions and were tested on consecutive days to reduce carryover effects and fatigue, on about the same time of the day, lasting a maximum of 1 h/day. For patients who still fatigued very fast, the additional clinical testing was postponed to a 3rd day. During testing participants were asked for any discomfort or motion sickness. Neither participants nor examiners were blinded due to the optical see-through device being used.

### Software Development

The testing environments were designed using the game engine development tool, Unity 3D (Version 2017.4). The five objects were created by 3D-scanning their real-life counterparts in order to achieve high visual fidelity. Object selection was based on its movement characteristics to cover a variety of different movement components, movement planes and grip formations (e.g., repetitive hammering with elbow flexion/extension using a cylindrical grip in the longitudinal plane). Three of the five gestures involved non-repetitive movements (water a plant, iron a blouse, open a lock), while the other two were repetitive gestures (screw in an electric bulb, hammer a nail). For this study we chose gestures performed without body contact because of the complexity of holographic animations performed on the body. Only the tools and not their corresponding counterpart were shown (i.e., the hammer, but not a nail, see Figure 2D). The dynamic version is based on recordings of real tool use movements with the same physical objects (including the recipient object) using motion capturing (Qualisys Inc., Gothenburg, Sweden). The gathered kinematic data were post-processed to handle noise. In the screen environment, the objects had to be adjusted in size in order to be properly displayed on the screen. In the HMD environment, we adjusted the objects' position in space to maintain the objects' real sizes. Further, the objects were oriented in space in a way that the tools' handle functioned as an easy to graspable stimulus (38). The full project code is available at GitHub https://github.com/Ninarohrbach/panto-holo, and a visualization of the object presentations can be found in the supplements (Supplementary Videos 2, 3).

### Remote Control System

Generally interacting with the HoloLens device as an experimenter is inconvenient, because one would need to put on the device for each single interaction. We solved this problem by using a web application to remotely control the HoloLens application (see Supplementary Video 1). The advantage of a web application is that it can be run on almost any device that has a web browser, e.g., smartphones. The complete system consisted of three components: The web application, a webserver and the HoloLens application. The HoloLens application was implemented using Unity 2017.4 using C++. A Firebase application was used as a web server and Polymer 2.0 was used for the front-end of the web application. This way, the experimenter could easily change the values (i.e., object 1–5, and mode “static”/“dynamic”) on the Firebase server in real-time. The same system was used for the screen environment, by running the Unity application on a laptop.

### Clinical Tests and Questionnaires

Prior testing, participants were asked questions regarding their sociodemographic background and previous HMD experience. The Mini Mental State Examination (MMSE) (39) was conducted to assess cognitive impairment. The Titmus Test (Stereo Optical Co., Chicago, IL) with its two sub-tests was administered to classify for the presence (i.e., House Fly test) and the quality of stereovision (i.e., Circles test). The Edinburgh Handedness Inventory (EDI) (40) was used to assess the dominance of a person's hand in everyday activities before the stroke. To evaluate manual dexterity, we conducted the Nine Hole Peg Test (NHPT) (41). For this purpose, the left (non-paretic) hand was tested twice using motion capture analysis and the mean time of two successful trials was computed (see “hand kinematics” in data analysis). Further, we examined the Motricity Index (MI) to evaluate the extent of the paralysis of the affected arm by assessing the strength (remaining force) of shoulder abduction, elbow flexion and finger griping (42). To diagnose for the presence of apraxia the Diagnostic Instrument for Limb Apraxia—Short Version (DILA-S) was used (13). Note, that the DILA-S was evaluated for patients with LBD and is applicable for patients with severe aphasia or neglect. At the end of each testing condition (i.e., four times), participants completed a slightly adapted presence questionnaire (43) (Supplementary Table 1).

## Data Analysis

### Scoring System

Supplementary Table 2 provides details on the scoring procedure. As the primary outcome parameter, a performance scoring was undertaken. For task evaluation we adapted the **Production scale** (PS) (13) in which four movement components were rated on a three-point scale resulting in a maximum score of 24 points per object and condition after three trials. Additionally, we applied the **Interaction scale** (IS) developed for the purpose of this study to investigate the participants' interaction with the different cues. With the standard pantomime procedure in clinical settings the examiner sometimes observes patients who seemingly try to interact with the presented item by reaching for and touching the depicted picture. One point per trial was given if participants actively tried to reach forward and grasp the virtual object or followed the movement, ending up with a maximum of three points per object and condition after three trials. Note that our experimental task and digital content do not require any interaction. Thus, the term “interaction” within this study does not reflect the overall accepted definition in the AR domain [for a recent review on immersive systems (44)].

Each participant's videotaped performance was viewed in its full length four times, once for each of the four movement parts. Two independent raters (NR, LL) scored the first 20 participants (10 patients, 10 controls) and critical aspects were discussed within the research team in a consensus meeting. Validating a certain percentage of the study sample by two independent evaluators is common and widely accepted practice e.g., 25% in (18) and (45). The inter-rater reliability of the pantomime scoring (400 data points for the Production and Interaction scale) and real tool scoring (50 data points) of the first ten healthy control subjects achieved large results for pantomiming (Kendall's Tau τ = 0.643 for Production; τ = 0.602 for Interaction) and real tool demo (τ = 0.862). After further refinement of the system, all data were scored and uncertainties were collaboratively discussed until the two raters met consensus.

### Statistical Analysis

All outcome variables were tested for normal distribution using Shapiro-Wilk's test. The statistical analysis included a *t*-test for age and non-parametric tests for sex, stereovision, MMSE and NHPT-time to determine if there were differences between the patient and the control group. For the pantomime performance (averaged score across all five objects for each of the four conditions) and the subjective experience of the presented objects (calculated mean score of presence data for each of the four conditions) a mixed repeated measures 2 × 2 × 2 ANOVA was conducted to determine whether any changes in the dependent variables (Production Scale, Interaction Scale) were caused by the between-subject factor *Group* (Stroke, Control), the within-subject factors *Environment* (Screen, HMD) and *Mode* (Static, Dynamic), or their interactions. We dealt with missing values (Production: 2.06%, Interaction: 2.14%) by imputing the mean performance value for the respective object and condition (46). Significant interactions, simple effects and main effects were followed-up with Bonferroni-adjusted pairwise *post-hoc* tests comparing the performance scores of the different visual cues. The achieved real tool scores were compared separately between groups using independent *t*-tests. They were further analyzed within each group, by comparing them with the means of the four combinations of the pantomime task using *t*-tests for paired samples. We calculated the performance effects, i.e., the environmental (HMD-Effect), the conditional (DYN-Effect) and the combined effect (HOLO-Effect) for both scales, defined as the following:

- HMD-Effect = Mean (HMD^Stat^, HMD^Dyn^) – Mean (Screen^Stat^, Screen^Dyn^)
- DYN-Effect = Mean (HMD^Dyn^, Screen^Dyn^) – Mean (HMD^Stat^, Screen^Stat^)
- HOLO-Effect = Mean HMD^Dyn^ – Mean (Screen^Stat^, Screen^Dyn^, HMD^Stat^)

We assessed the relationship of the Production and Interaction scores within each group using Spearman's rank correlation (r_s_). Further, the performance effects were correlated with the clinical data to test whether the timing of stroke onset, mental capacity, manual dexterity, stereovision or apraxia affect pantomime of tool use using Pearson's r or Spearman's correlation. The relationship between presence and pantomiming was analyzed for each condition within the patient group. For significant correlations, the magnitude was classified considering the following categories: |*r*| ≥ 0.10 = small, |*r*| ≥ 0.30 = medium and |*r*| ≥ 0.50 = large (47). Data analysis was carried out in SPSS (version 26), and the level of significance was established at a 0.05 alpha-level (two-sided).

### Hand Kinematics

**Figure 3:**
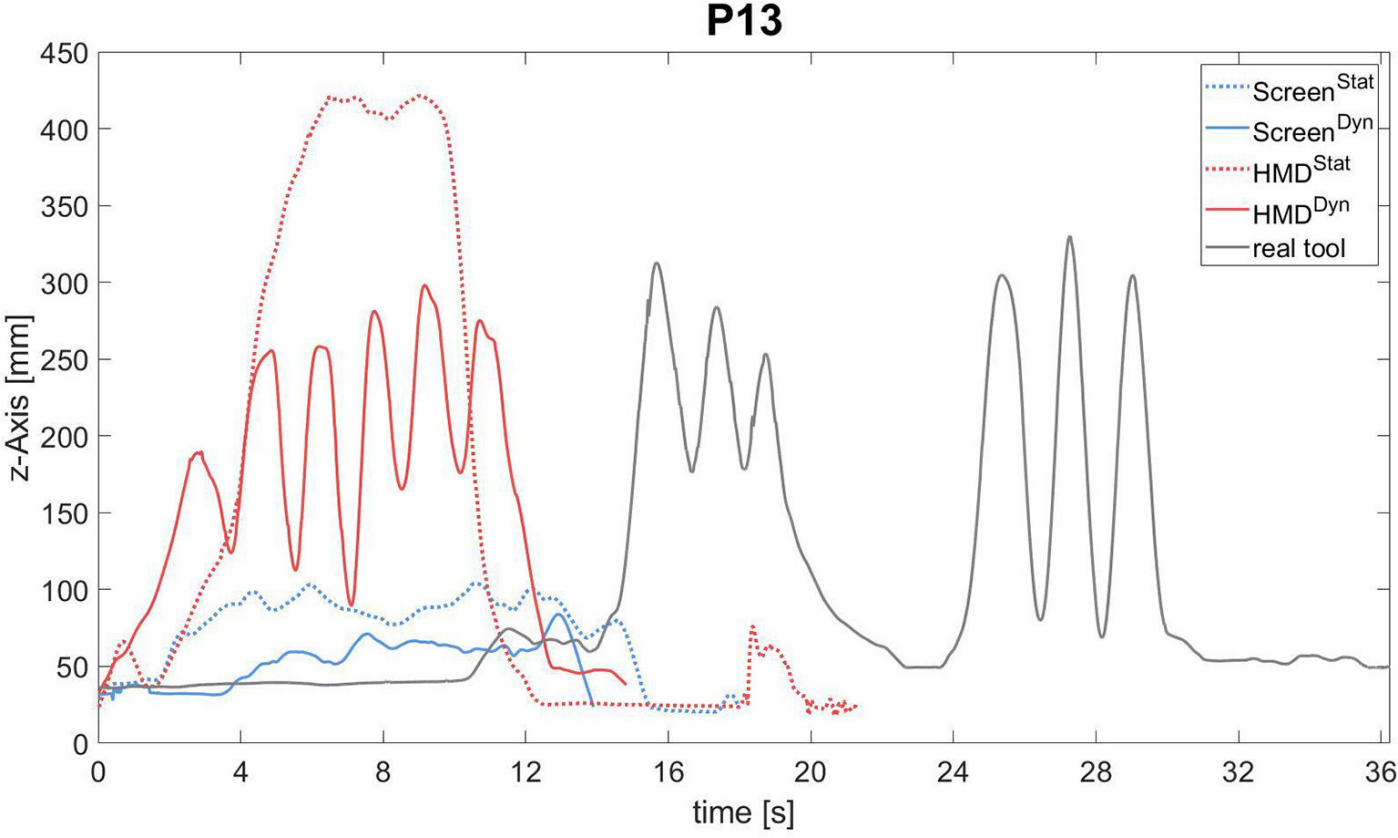
Trajectories of hand movements in patient P13 attempting to pantomime the typical use of a hammer during the different experimental conditions is illustrated: Screen^Stat^ (blue line), Screen^Dyn^ (blue dotted line), HMD^Stat^ (red line), HMD^Dyn^ (red dotted line), real tool (gray line). The complete trajectory along the z-Axis in (mm) during the third of three trials is always shown.

In addition, we recorded hand movements (a spherical marker attached to the subject's left back of the hand) using motion capturing. Movements were recorded by three cameras (Oquus, Qualisys Inc., Gothenborg, Sweden) and a sample rate of 120 Hz. The kinematic approach served as an objective and sensitive analysis to evaluate the NHPT data and to provide an additional visual illustration to our qualitative findings. Based on the performance results, the patient with the strongest HOLO-Effect (see statistical analysis for further specification) was chosen for further kinematic analysis. Post-processing of the hammering performance (repetitive up and down movement) of P13 was performed using MATLAB R2018b (MathWorks, Natick, MA, USA). We determined the starting and the ending time points by calculating the overall marker velocity in 3D space and thresholding it at v_th_ = 0.012 [m/s]. The vertical axis of the movement was extracted and plotted for visualization (Figure 3).

## Results

### Participant Demographics

Participant characteristics and patient-specific information are provided in Tables 1, 2. All but one patient (P23) showed signs of apraxia in at least one of the DILA-S sub-tests (Supplementary Table 3), with most patients being affected in the Imitation of gestures (meaningless: 95%, meaningful: 67%), in the Pantomime task (Production: 76%, Execution: 71%) and in the Naturalistic Action Task (NAT: 62%). While the majority of patients had at least mild problems in the Familiar Tools Task (FTT; Selection: 33%, Production: 67%, Execution: 62%) they were less frequently affected in the Novel Tools Task (NTT; Selection: 52%, Production: 29%, Execution: 29%).

**Table 1:** Participant's demographics and clinical characteristics.

	**LBD (*N = 21*)**	**Controls (*N* = 23)**	**Between-Group Comparisons**
Sex: male/female	10/11	10/13	t_(42)_ = −0.988, *p* = 0.329
Age: mean years (range)Adverse events, side effects^*^: yes/no	69.81 (41–91)0/21	65.87 (40–91)0/23	*U* = 231.5, *Z* = −0.272, *p* = 1.0
EDI: right/left/both	20/0/1	23/0/0	
Education level^**^: low/middle/high	8/8/4	6/7/10	
Experience with HMD: yes/no	0/21	0/23	
Etiology: Ischemic infarct/ICB	18/3	NA	
Aphasia^***^: yes/no	15/6	NA	
MMSE: mean (range)	21.25 (14–28), *N* = 16	28.83 (24–30)	*U* = 8.500, *Z* = 34.6, *p* <0.001
MI: mean (range)	52.6 (0–100)	NA	
Neglect^****^: yes/no	6/15	NA	
NHPT: mean time in seconds (range)	47 (26–140)	24.5 (18.5–44)	*U* = 445.00, *Z* = 42.5, *p* <0.001
Titmus Test			
House Fly: stereovision given (yes/no)	13/6	23/0	*U* = 138.0, *Z* = −3.151, *p = 0*.002
Circles: ≤ /> 100 arc/sec	2/16	17/6	*U* = 77.0, *Z* = −3.953, *p* <0.001
Time since event: mean duration in days (range)	250,7 (11–1,933)	NA	
Visual aids during testing: yes/no	12/9	21/3	

*EDI, Edinburgh Hand Inventory; HMD, Head Mounted Display; ICB, Intracranial bleeding; LBD, Left Brain Damage; MMSE, Mini Mental State Examination; MI, Motricity Index; NA, Not applicable; NHPT, Nine Hole Peg Test (left hand); t, t test for independent samples; U, Mann-Whitney-U-Test,*

* *based on verbal reports,*

** *Education level: low = secondary school, middle = intermediate school =, high = high school or higher,*

*** *based on Aachen Aphasia Test (AAT) analysis description, i.e., a combination of the subscales Token Test and written language,*

**** *based on different severity levels assessed with different assessments; information provided by neuropsychologists out of a test battery including several paper-pencil tests*.

**Table 2:** Patient's characteristics.

**ID**	**Sex**	**EDI**	**Age (y)**	**ICD-10**	**Etiology**	Stage^*^	**Neglect**	**Aphasia**	**MI**	**NHPT (t)**	**Stereovision**	**MMSE**
P01	M	right	64	I61.2	ICB	sub-acute	no	Yes	76	42,44	intact	NA
P02	M	right	51	I61.0	ICB	sub-acute	yes	Yes	11	27,77	NA	19
P03	F	right	85	I63.4	Infarct	sub-acute	no	Yes	77	30,51	NA	25
P04	F	right	71	I63.5	Infarct	sub-acute	no	Yes	0	26,18	intact	21
P05	M	right	41	I63.3	Infarct	chronic	no	Yes	0	28,89	impaired	NA
P06	F	right	89	I63.4	Infarct	sub-acute	no	Yes	0	58,38	impaired	NA
P07	M	right	64	I63.4	Infarct	sub-acute	no	Yes	66	30,18	intact	23
P08	M	right	69	I63.2	Infarct	sub-acute	no	Yes	100	81,78	intact	24
P09	M	right	80	G82.29	Infarct	sub-acute	no	No	76	39,50	intact	24
P10	F	right	90	I63.4	Infarct	sub-acute	no	No	88	42,12	impaired	17
P11	M	both	74	I63.4	Infarct	sub-acute	no	Yes	77	39,04	intact	19
P13	F	right	61	I63.4	Infarct	chronic	yes	Yes	78	139,66	impaired	NA
P14	F	right	54	I63.0	Infarct	sub-acute	yes	No	39	59,01	impaired	26
P16	F	right	85	I.63.4	Infarct	chronic	no	Yes	0	61,05	intact	NA
P17	M	right	83	I.63.1	Infarct	sub-acute	no	Yes	100	42,78	intact	14
P18	F	right	72	I63.0	infarct	sub-acute	no	No	0	25,94	intact	19
P19	M	right	65	I63.4	Infarct	sub-acute	yes	Yes	100	68,68	impaired	16
P20	M	right	56	I61.1	ICB	sub-acute	no	Yes	83	28,79	intact	28
P21	F	right	91	I63.5	Infarct	chronic	no	No	100	37,87	intact	21
P22	F	right	79	I67.88	Infarct	sub-acute	yes	Yes	34	52,03	intact	25
P23	F	right	42	I63.5	Infarct	chronic	yes	No	0	26,62	intact	19

*Due to communication problems, not all patients could be tested for stereovision and cognition, but comprehension was sufficient to follow task instructions and all patients were able to complete the AR-testing.*

*EDI, Edinburgh Hand Inventory; F, Female; ICB, Intracranial bleeding; ICD-10, International Classification of Diseases-Tenth Revision; M, Male; MMSE, Mini Mental State Examination; MI, Motricity Index; NA, Not applicable; NHPT(t), Nine Hole Peg Test (time in seconds, with left hand).*

* *Stage: Sub-acute= <6 months, chronic: >6 months*.

### Performance Results

**Figure 4:**
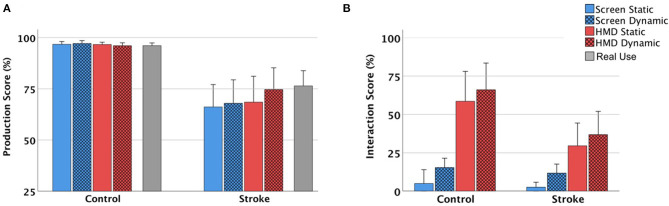
**(A,B)** Results of the pantomime performance of the control group (left) and stroke group (right); Mean and 95% confidence interval values for the interactions of Environment (HMD/Screen) and Mode (static/dynamic) in **(A)** Production scale and **(B)** Interaction scale are reported.

**Figure 5:**
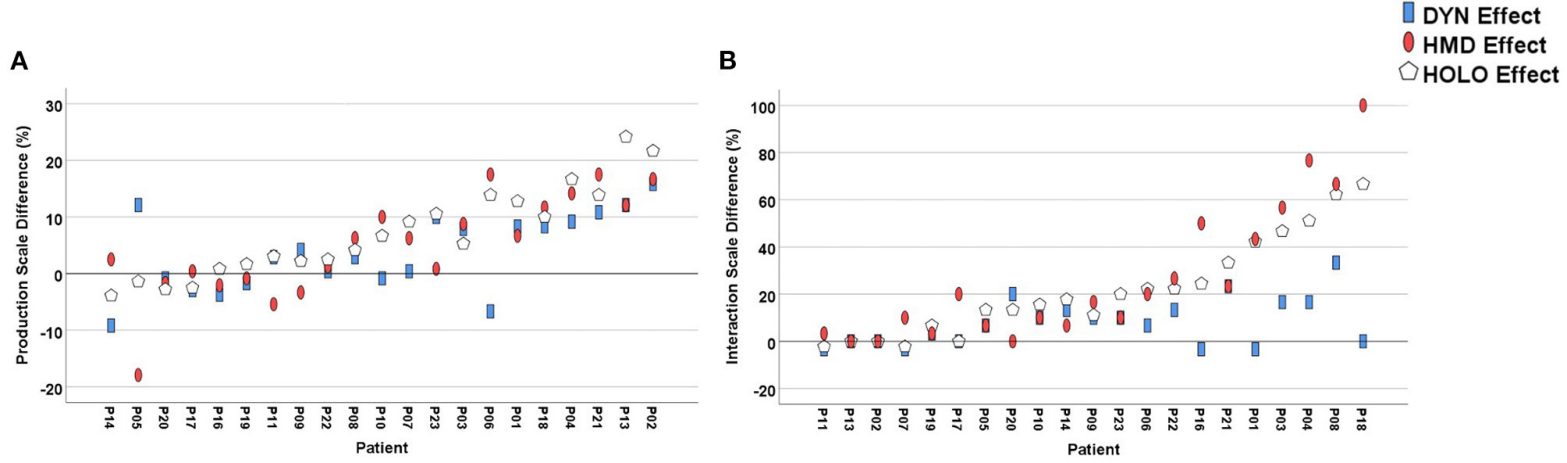
Conditional (DYN), environmental (HMD) and combined (HOLO) effects of individual patients displayed in **(A)** Production Scale and **(B)** Interaction Scale.

Figure 4 displays the performance scores of both groups of the Production and Interaction scales, and Table 3 shows the ANOVA results respectively. The individually achieved environmental (HMD-Effect), modal (DYN-Effect) and combined (HOLO-Effect) effects in patients are visualized in Figure 5. During HMD trials, the key was not visible for three patients (P1&P6: Key_HMD^Stat^, P1&P16: Key_HMD^Dyn^), and in another patient (P21) the Screen^Stat^ condition was not videotaped. Overall, we had a total of 26 missing data points out of 1,260 observations on the Production scale (2.06%) and 9 out of 420 on the Interaction scale (2.14%), respectively.

**Table 3:** ANOVA summary for production scale, interaction scale and sense of presence.

**Production Scale**	**Statistical parameters**					
	***F*** **(df)**		***p***		**Effect size** $\eta_{\text{p}}^{2}$	
*Group*	*F*_(1, 42)_ = 28.6		<0.001		0.405	
*Environment*	*F*_(1, 42)_ = 4.9		0.031		0.106	
*Mode*	*F*_(1, 42)_ = 6.2		0.017		0.129	
*Group × Environment*	*F*_(1, 42)_ = 8.2		0.007		0.163	
*Group × Mode*	*F*_(1, 42)_ = 6.8		0.012		0.140	
*Environment × Mode*	*F*_(1, 42)_ = 1.7		0.203		0.038	
*Group × Environment × Mode*	*F*_(1, 42)_ = 4.5		0.039		0.097	
	**Healthy subjects**		**Patients**			
	***F*** **(df)**	***p***	**Effect size** $\eta_{\text{p}}^{2}$	***F*** **(df)**	***p***	**Effect size** $\eta_{\text{p}}^{2}$
*Environment*	*F*_(1, 22)_ = 1.9	0.176	0.082	*F*_(1, 20)_ = 6.2	0.021	0.238
*Mode*	*F*_(1, 22)_ = 0.05	0.826	0.002	*F*_(1, 20)_ = 6.5	0.019	0.244
*Environment × Mode*	*F*_(1, 22)_ = 2.3	0.144	0.095	*F*_(1, 20)_ = 2.9	0.103	0.127
**Interaction Scale**						
	***F*** **(df)**		***p***		**Effect size** $\eta_{\text{p}}^{2}$	
*Group*	*F*_(1, 42)_ = 6.5		0.014		0.135	
*Environment*	*F*_(1, 42)_ = 55.8		<0.001		0.570	
*Mode*	*F*_(1, 42)_ = 11.3		0.002		0.213	
*Group × Environment*	*F*_(1, 42)_ = 6.1		0.017		0.127	
*Group × Mode*	*F*_(1, 42)_ = 0.03		0.862		0.518	
*Environment × Mode*	*F*_(1, 42)_ = 0.43		0.518		0.010	
*Group × Environment × Mode*	*F*_(1, 42)_ = 0.01		0.932		0.000	
	**Healthy subjects**		**Patients**			
	***F*** **(df)**	***p***	**Effect size** $\eta_{\text{p}}^{2}$	***F*** **(df)**	***p***	**Effect size** $\eta_{\text{p}}^{2}$
*Environment*	*F*_(1, 22)_ = 39.9	<0.001	0.645	*F*_(1, 20)_ = 17.7	<0.001	0.470
*Mode*	*F*_(1, 22)_ = 4.16	0.052	0.159	*F*_(1, 20)_ = 13.5	0.001	0.403
*Environment × Mode*	*F*_(1, 22)_ = 0.20	0.657	0.009	*F*_(1, 20)_ = 0.277	0.605	0.014
SENSE OF PRESENCE^*^						
	***F*** **(df)**		***p***		**Effect size** $\eta_{\text{p}}^{2}$	
*Group*	*F*_(1, 34)_ = 0.120		0.731		0.004	
*Environment*	*F*_(1, 34)_ = 27.9		<0.001		0.450	
*Mode*	*F*_(1, 34)_ = 0.28		0.601		0.008	
*Group × Environment*	*F*_(1, 34)_ = 5.5		0.025		0.139	
*Group × Mode*	*F*_(1, 34)_ = 0.48		0.494		0.014	
*Environment × Mode*	*F*_(1, 34)_ = 0.02		0.886		0.001	
*Group × Environment × Mode*	*F*_(1, 34)_ = 0.27		0.605		0.008	

* *A few participants did not answer Q3 (HMD^Stat^: C8, C9, P20; HMD^Dyn^: C9, P20), herein, we imputed the mean within each group. Eight patients did not or only partially fill in the presence questionnaire (P4, P6, P10, P12, P13, P14, P15, P16), thus, we included 36 data sets in the mrANOVA (Controls n = 23, Patients n = 13)*.

### Production Scores

On the Production scale, a significant main effect of *Group* with overall higher scores in controls (Figure 4A) indicates that healthy subjects performed significantly better than patients (*MD* = 6.5; 95%-CI [4.1,8.9], *p* < 0.001). Further, we found significant main effects of *Environment, Mode* and significant interactions between *Environment* × *Group, Mode* × *Group*, and *Environment* × *Mode* × *Group*, but not between *Environment* × *Mode* (Table 3).

Next, we analyzed the different combinations within each group separately. Control participants reached almost maximum scores independent of the presented stimuli (*M* = 23.2, *SD* = 0.64 [21.4,23.9] with no significant effects or interactions (*p* > 0.144). In patients, we found a statistically significant effect of *Environment* and of *Mode*, but not between *Environment* × *Mode*. Bonferroni-adjusted pairwise comparisons indicate a better performance with the help of holographic (−1.2; 95%-CI [−2.1,−0.19], *p* = 0.021) or dynamic cues (−0.91; 95%-CI [−1.7,−0.16], *p* = 0.019).

### Interaction Scores

We found a significant main effect of *Group* on the Interaction scale, suggesting that healthy subjects interacted significantly more with the presented stimuli (0.48; 95%-CI [0.10,0.86], *p* = 0.014; Figure 4C). Similar to the Production scores, we found significant main effects of *Environment* and of *Mode*, and a significant *Environment* × *Group* interaction which was driven by higher means in the HMD Environment in controls (Screen: 0.30; 95%-CI [0.13,0.47], HMD: 1.9; 95%-CI [1.4,2.4] compared to patients (Screen: 0.21; 95%-CI [0.11,0.32]; HMD: 1.0; 95%-CI [0.56,1.4]. All remaining interactions were non-significant (*p* > 0.518, Table 3).

In both groups, there was a significant effect of *Environment*, suggesting stronger effects of holographic than screen-based cues (Patients: −0.79; 95%-CI [−1.2,−0.39], *p* < 0.001; Controls: −1.6; 95%-CI [−2.1,−1.1], *p* < 0.001). A significant effect of *Mode* in patients and a borderline significant effect of *Mode* in controls (*p* = 0.054) point toward a higher effect of dynamic than static cues (Patients: −0.24; 95%-CI [−0.38,−0.11], *p* = 0.001; Controls: −0.27; 95%-CI [−0.54,0.005], *p* = 0.054).

### Correlations Between Production and Interaction Scores

We found medium to large significant correlations between the Production and Interaction scores. In patients, higher interactions with animated screen-based objects were significantly associated with a better performance (Screen^Dyn^ r_s_ = 0.699, *p* < 0.001). In controls by contrast, when the interaction with static holographic items increased, the performance decreased (HMD^Stat^ r_s_ = −0.537, *p* = 0.008). All other correlations were non-significant (Supplementary Table 4).

### Real Tool Comparison

Patients had significant problems demonstrating the real tool use (*M* = 18.3, *SD* = 3.9) compared to controls [*M* = 23, *SD* = 0.74, *t*_(42)_ = 5.7, *p* < 0.001]. In healthy subjects, all pairwise comparisons were non-significant (*p* > 0.208). In patients, there was a significant difference between real tool use (*M* = 18.3, *SD* = 3.9) and the Production scores achieved in Screen^Stat^ [*M* = 15.9, *SD* = 5.8, *t*_(20)_ = 3.7, *p* = 0.001], Screen^Dyn^ [*M* = 16.3, *SD* = 6.0, *t*_(20)_ = 3.0, *p* = 0.007], and HMD^Stat^ environments [*M* = 16.5, *SD* = 6.6, *t*_(20)_ = 2.4, *p* = 0.027]. In contrast, there was no difference between real tool use and the Production scores observed in the HMD^Dyn^ environment [*M* = 17.9, *SD* = 5.6, *t*_(20)_ = 0.75, *p* = 0.461), suggesting that the performance was best when either receiving dynamic holographic cues or when demonstrating real tool use (Figure 4).

### Correlations Between Clinical Data and Pantomime Performance Effects

On the Production scale, a higher DYN-Effect was associated with a higher Circles score (r_s_ = 0.524, *p* = 0.026), a higher NHPT time (r_s_ = −0.695, *p* < 0.001), and a lower NTT Selection score (r_s_ = −0.498, *p* = 0.021). On the Interaction scale, a lower DYN-Effect goes along with a lower MMSE score (r = 0.550, *p* = 0.027), and with worse performances in object-interaction tasks (FTT Production r_s_ = 0.510, *p* = 0.018; NAT r_s_ = 0.546, *p* = 0.013). Further, a non-significant trend between stereovision and the HOLO-Effect^IS^ points toward more frequent interactions with animated holographic items when a higher quality in stereovision is given (r_s_ = 0.449, *p* = 0.061). All other correlations between any of the calculated effects and the clinical tests failed to reveal statistical significance. See Supplementary Tables 5, 6 for correlations with clinical data and DILA-S results.

### Kinematic Analysis

Kinematic analyses were run in order to visualize the qualitative findings. Figure 3 exemplarily depicts the kinematic analysis for patient 13 who experienced the strongest “HOLO-Effect” based on the results of the performance scoring (Figure 5). The complete trajectory along the z-Axis in (mm) of the most successful version of each condition is always shown (here, the third of the three trials, respectively). In real tool demonstration she failed during the first (Production: 0 points) and second attempt (Production: two points for grip formation when grasping the hammer), but she managed to perform a nice hammering movement (Production: seven points, −1 because of a distorted movement orientation) after some hesitation in her last trial (“conduite d'approche,” after all it still took her 10 s to initiate the action). All her attempts to pantomime hammering in Screen^Stat^, Screen^Dyn^, and HMD^Stat^ were characterized by “toying” (Production: zero points in all conditions, respectively). In the HMD^Dyn^ condition by contrast, she presented clear up- and downwards hits with the support of the animated holographic hammer during her second and third attempts (Production: seven points in both attempts; −1 because of distorted grip formation). Note, P13 was randomized to receive HMD-based cues first, followed by screen-based cues on day 2. The corresponding video can be found in the supplements (Supplementary Video 4). The analyses demonstrated that the qualitative findings can be verified by kinematic trajectories showing a clear improvement with HMD^Dyn^ support (HOLO-Effect).

### Sense of Presence

The statistics is shown in Table 3. The two groups did not differ significantly (*p* = 0.731). We found a significant main effect of *Environment* and a significant *Environment* × *Group* interaction, which was driven by a higher sense of presence in the HMD than in the screen environment (Controls^Screen^: 2.9, 95%-CI [2.4,3.4], Controls^HMD^: 4.7, 95%-CI [4.4,4.9], Patients^Screen^: 3.3, 95%-CI [2.7,4.1], Patients^HMD^: 4.1, 95%-CI [3.7,4.3]). Realness of the presented objects was rated as high in the screen environment (*M* = 3.4, *SD* = 1.8) and very high in the HMD environment (*M* = 4.8, *SD* = 1). While spatial presence was judged low in the screen environment (*M* = 2.4, *SD* = 1.9) it was rated as very high in the HMD environment (*M* = 5.1, *SD* = 1). Perceptual stress was perceived as moderate in both environments (Screen *M* = 3.4, *SD* = 1.2; HMD *M* = 3.4, *SD* = 1.4). All other effects and interactions were non-significant (*p* > 0.494).

### Correlations Between Presence and Pantomiming

We found a significant correlation between presence and HMD^Dyn^ Production results (r = 0.534, *p* = 0.049), suggesting that as the sense of presence increases with animated holograms, so does the performance. All other correlations were non-significant (Supplementary Table 7).

## Discussion

In this study the effects of pantomiming with visual feedback provided in different environments (Screen vs. HMD) and different modes (static vs. dynamic) and the impact of presence in each condition were compared. Age-matched control participants performed as expected, close to ceiling in all conditions and significantly better than patients. In contrast, the patients' performances were dependent upon the type of visual feedback given. As hypothesized, patients achieved significantly higher scores when they received holographic (HMD-Effect) or dynamic cues (DYN-Effect). Despite not reaching the level of significance, best results were observed with dynamic holograms (HOLO-Effect, Figure 5A). Impressively, single patients improved their overall performance of up to 24% with this form of visual support. The kinematic analysis of one particularly impressive patient (P13), who failed in all conditions except when cued with animated holograms, is shown in Figure 3 and Supplementary Video 4.

A key finding within this study is that pantomiming tended toward the real tool demonstration performance with the support of visual stimuli of increasing salience (Figure 4A). It has been hypothesized that different representations underline pantomimed actions and real tool use, with pantomimes serving communication (when trying to enable others to recognize the pretended actions) while real tool actions being instrumental (10, 17, 21, 48). One possible explanation for behavioral improvement when presented with salient stimuli is that the provided holographic cues facilitated compensatory action simulation processes by triggering activities in relevant cortical areas for pantomime of tool use (49). Lesion symptom mapping studies show that defective pantomime of tool use is associated with damage in left ventro-dorsal regions (14, 50, 51), with communicative aspects being related to rather anterior regions in the inferior frontal cortex, and aspects related to motor cognitive movement production being rather associated with posterior regions in the network (5). The latter lesion correlates in left parietal regions are in line with those reported to go along with deficient demonstration of tool use (52). Given the salient nature of holographic presentations of familiar objects one may hypothesize that more specific neural responses in ventral visual streams have been elicited by object recognition processes. Present information about the object may help to specify potential actions by narrowing down action opportunities supported by rather posterior and dorsal regions. Perhaps these processes elicited by the salient cues may help channeling higher-order functions such as attention and reduce the load on action simulation processes in a left fronto-temporo-parietal network. In line with this idea, the visual streams in the ventral and dorsal cortex, that are responsible for perceiving and interacting with common objects in the three-dimensional space, have been shown to respond similarly in AR tasks as compared to real-world tasks (53). Thus, one reason for improved pantomiming might be that the increased saliency in visual input has shifted the pantomime actions from communicative gestures to rather instrumental actions.

Clearly, a strength of this study lies in the design of holograms by 3D-scanning the original tools and recording its real use. The induced sense of presence was significantly higher in HMD than in screen environments, and in the HMD^Dyn^ environment pantomiming improved significantly with higher presence ratings. The realness and high spatial presence evoked by our holograms may have made pantomiming less symbolic as it was rather influenced by the strong external cues. Further, it has been shown that apraxics have deficits in intrinsic coordinate control (11, 22). In such, participants might have extrinsically coordinated their movements in reference to the dynamic or holographic objects. The context factors in the HMD environment, e.g., the orientation in space (designed in a way to invite the participant to reach for it) and the real-sized holograms might have reduced the opportunities of grip formation and movement orientation, thereby limiting the degrees of freedom. Moreover, the structural and texture information, including light reflections, given in our holograms could have helped patients (37). These details became even more extensive in HMD^Dyn^ conditions, offering different perspectives, such as the view of the bottom of the watering can when it is moved. For instance, some patients showed clear difficulties in spatial orientation in screen conditions, but the holographic presentations helped them orientating in space correctly.

Lastly, the dynamic presentation in both environments might have attracted more attention and have had a more prompting character stimulating the correct movement content (20). In this regard, we observed individual patients trying to copy the shown movements, e.g., by following the rhythmic beat of hammering. In neuroimaging studies investigating healthy people, a larger response in the lateral temporal cortex relative to the ventral cortex has been shown when dynamic compared to static humans and tools are viewed, suggesting the lateral temporal cortex to be responsible for complex motion processing (54). Potentially, the moving cues enhanced the activity in the lateral temporal cortex which may have been integrated into the perception-action network processing pantomimes.

This can be partially supported by the Interaction scores, showing significant higher object interactions in HMD or DYN conditions. In patients, higher interactions during the Screen^Dyn^ condition even significantly correlated with increased Production scores, which indicates an added value of dynamic cues in screen-based systems. In addition, patients with a higher quality in stereovision, a better manual dexterity and worse mechanical problem solving benefit more from dynamic cues. One possible explanation is that patients with mechanical problem solving deficits may profit from the increasing visual and semantic information consistent with the task provided by the three-dimensional cues from the HoloLens (e.g., when focusing perception on the best suited affordances to solve the task, here the correct representation of the moving tool). Indirectly, this could be taken as an indicator of an important role of mechanical problem solving in tool use behavior and would therefore be in line with the reasoning-based approach to human tool use (23, 55, 56).

Nevertheless, correlations between Interaction and Production scores during HMD conditions did not become significant (*p* > 0.22). In contrast, and probably even more striking, the patients who experienced the strongest HOLO-Effects on the Production scores (P13, P02) did not interact with the given cues at all (Figure 5). Moreover, in healthy subjects the interactions with static holograms even negatively influenced performance, in a way that they changed their motor behavior resulting in unnatural, error-loaded movements when trying to reach for holograms. Potentially, these participants got distracted from the actual task by volitionally directing their attentional focus on the salient cues (36), resulting in more errors. That is, consistent with the results of a feasibility study on AR-based ADL support, the unnatural interaction with holographic animations that impaired the performance by requesting its own resources (57). We would have expected higher presence to result in more interactions with the virtual objects. However, we did not find a significant correlation which can be explained by the experimental task design not *requiring* any real interaction. Still, at this point it remains unclear why some participants were very responsive to the stimuli (such as P18, who interacted with holograms in 100% of the HMD conditions), while others seemed not to respond at all (Figure 5). The interaction with dynamic objects was higher in controls as well as in patients with a higher mental state, a better FTT Selection and NAT score. Possibly, unimpaired people are more prone to interacting with holograms because they have more cognitive resources to focus on the augmented information, but this hypothesis has to be further investigated.

Another likely explanation for the improvements is that both the dynamic and holographic information provided error signals for the perceptual-motor system as suggested by Jax et al. (11). While patients with apraxia often struggle in movement preparation (i.e., planning) the adjustment of the movement plan (i.e., online correction) is often intact (22). Similar to reports of Jax and colleagues (11) about the observed “conduit d'approche” in some patients, we also noted an increase in accuracy after multiple repetitions. Patients might have visually recognized their incorrect movements and tried to more closely approximate the correct action represented by the animated holograms.

### Limitations

The psychometric properties of the applied Presence questionnaire (43) have not yet been validated in the stroke population or in patients with cognitive limitations. Unfortunately, eight patients failed to fill in the questionnaire, which indicates that it may not be the best measure to assess presence in this population. Besides a need of alternative questionnaires, the integration of objective measures (e.g., eye movements) is worth further investigation. In HoloLens 2nd generation, the feature of eye-tracking is incorporated offering an easy way to analyse visual attention based on eye movements, to assess salience and to identify the user's intention (35) and areas of interests (23). Indeed, while spatial attention is a major mechanism for saliency detection, patients with visuo-spatial or attentional deficits might not be able to focus their limited perceptual resources on the holograms. In this study, patients with a higher quality in stereovision had a higher DYN-Effect on the Production scale and a trend points toward an association of higher stereovision and interactions with animated holograms. We cannot rule out that some patients have been unable to see the holograms as intended and thus, have not benefited from its salient contextual information.

The technical presentation of realistic holograms also had its short-comings. In particular, some patients were unable to detect the key, possibly because it was displayed too close to the user and might have been overlooked because of not being visually distinct enough from its surrounding. On the other hand, participants criticized the holographic watering can appearing too far away in order to grasp for it, which was necessary to enable real-size presentations in the HoloLens. This illustrates the difficulty in finding the optimal zone for hologram positioning in experimental research, especially with the current technological limitations (e.g., limited field of view). The fact that the dynamic features had no significant impact on presence ratings may be due to these technological constraints (28).

The predefined eligibility criteria within the present study were quite broad. Consequently, we included patients in the subacute as well as in the chronic stage, patients with and without a diagnose of neglect, aphasia or cognitive decline, but did not adjust for these possible confounding factors. At the moment we are therefore not able to give differential recommendations to patients early and late after stroke. In addition, the effect of cues may have been underestimated in some patients if aphasia, neglect or attention deficits had deteriorated task understanding or stimulus perception. Further and in line with recent recommendations on post-stroke rehabilitation trials (58), we ensured an aphasia and neglect friendly testing (by following the DILA-S recommendations), which improved our recruitment rate and increases the generalizability of our results.

### Outlook

Apraxia is a major predictor of poor functional performance in ADL and of increased dependence on caregivers. To date, effective rehabilitation strategies are still limited (9, 59) and mainly include compensatory approaches, such as strategy training (8, 60), errorless learning (61), behavioral training (62) or task-specific and meaningful training (63). In recent years, technology-based approaches facilitating single-tool use and multistep actions have been proposed as promising strategies (9, 64). AR technology has already found its way into a large field of applications, where holographic elements enrich the perception of the real environment, e.g., by providing cognitive support during different tasks (65). In the wide field of rehabilitation, AR will introduce new pathways for therapeutic or assistive approaches with the potential of providing an engaging and motivating training environment (31), improving physical outcomes when applied as an adjunct therapy (29), supporting mental rehabilitation (44) or cognitive rehabilitation (57, 66). Based on our findings, we envision HMD-based AR systems to assist patients in their ADLs in the future, thus maintaining autonomy. The advantages of wearable cognitive support systems over existing screen-based approaches (66, 67) are having both hands available for interactions with the physical environment while still being able to move flexibly from one place to another. In this regard, we see two main application areas where AR can be used: (1) as a supportive training tool to facilitate performance improvement and (2) as a (well-controllable) diagnostic research tool to further examine the role and importance of different modes and types of visual cues and to identify predicting variables.

While we showed that holograms can attract attention (e.g., by being visually salient) and improve performance, they can potentially also distract from the real activity and may require voluntary effort to redirect the attention to the physical objects (36). The objects within this study were displayed in a left handed setting (Figure 2D) and the holographic cues were aligned in space to invite the participant to reach for it as it was shown that the perception of affordances (here the orientation of the tools in space) influences the motor response that is best suited for interacting with the target object (23, 56, 68). In future trials on real tool support however, we recommend to place cues in a non-reachable zone because no interaction with holographic but rather real objects is desired. Besides, AR supported manual task guidance inside the peripersonal space is associated with vergence-accomodation-conflict (e.g., when the virtual content is inconsistent with the real world) and focus-rivalry (e.g., when simultaneously focusing on real and virtual content). These common perceptual conflicts experienced in artificial environments may impair the performance due to visual fatigue and mental workload, especially with increased task difficulty as recently suggested by preliminary data on EEG recordings during AR use (69).

Future experiments should investigate whether a further increase in visual fidelity and contextual information will lead to even better results (e.g., by adding the target item or illustrating a holographic hand correctly performing the action). Indeed, findings from a recent eye-tracking study analyzing the visuo-perceptual context within a virtual scene show that thematically consistent object-tool pairs (e.g., hammer and nail) can have a facilitating influence on visual attention (23). In addition, audio-visual complexity does provide opportunities to enhance individual meaning, salience and authenticity (70–72).

## Conclusion

This study was the first to explore the effect of dynamic holographic cues on pantomiming in LBD patients. We provide first knowledge about which type of AR cue might be most beneficial in supporting patients with apraxia, present current limitations and give suggestions for further research. Specifically, studies are necessary to characterize the conditions that lead to optimal motor behavior in augmented environments, and to identify responders and factors that increase the potential effects of this new form of support. With further technological achievements (65) we believe this new approach to positively impact the rehabilitation process of patients with apraxia.

## Data Availability Statement

The datasets generated for this study can be found in online repositories. The names of the repository/repositories and accession number(s) can be found below: Center for Open Science (COS) Open Science Framework (OSF), https://osf.io/uakw2/?view_only=a55698fafb6541f7878284bab64e940c.

## Ethics Statement

The studies involving human participants were reviewed and approved by the Ethics Committee of the Medical Faculty of the Technical University of Munich (reference number 175/17S). The patients/participants provided their written informed consent to participate in this study.

## Author Contributions

NR and JH: conceptualization, methodology, and formal analysis. NR and AT: software and visualization. NR and LL: validation. NR, LL, and KJ: investigation. NR: data curation and writing – original draft preparation. CK, JR, KJ, and JH: writing – review & editing. CK: project administration. CK and JH: supervision. JR, KJ, and JH: resources. NR, CK, and JH: funding acquisition. All authors contributed to the final draft of the manuscript, read, and approved the final manuscript.

## Conflict of Interest

The authors declare that the research was conducted in the absence of any commercial or financial relationships that could be construed as a potential conflict of interest.

## Publisher's Note

All claims expressed in this article are solely those of the authors and do not necessarily represent those of their affiliated organizations, or those of the publisher, the editors and the reviewers. Any product that may be evaluated in this article, or claim that may be made by its manufacturer, is not guaranteed or endorsed by the publisher.

## Abbreviations

ADL: Activities of Daily Living
AR: Augmented Reality
DILA-S: Diagnostic Instrument for Limb Apraxia &#8211
Short Version
Dyn: Dynamic
EDI: Edinburgh Handedness Inventory
LBD: Left Brain Damage
MI: Motricity Index
MMSE: Mini Mental State Examination
NNPT: Nine Hole Peg Test
Stat: Static.
