# Supplementary material for: Improvement of Apraxia With Augmented Reality: Influencing Pantomime of Tool Use via Holographic Cues
Source: Front Neurol. 2021 Aug 26;12:711900. doi: 10.3389/fneur.2021.711900 (PMC8427527; doi:10.3389/fneur.2021.711900)
Supplement: Supplementary file 1 [file Table_1.DOCX]

Supplementary Tables

**Table 1. Sense of presence questionnaire**

| **Question** | **Theme** |
| --- | --- |
| Q1: Was watching the virtual objects just as natural as watching the real world? | Realness |
| Q2: Did you have the impression that you could have touched and grasped the virtual objects? | Realness |
| Q3: Did the virtual objects appear to be (visualized) on a screen, or did you have the impression that they were located in space? | Perceptual stress |
| Q4: Did you have the impression of seeing the virtual objects as merely flat images or as three-dimensional objects? | Perceptual stress |
| Q5: Did you pay attention at all to the difference between real and virtual objects? | Spatial presence |
| Q6: Did you have to make an effort to recognize the virtual objects as being three-dimensional? | Spatial presence |

Note: The questionnaire consisted of a series of six questions that were answered on a 7-point Likert scale to gauge the participant´s sense of presence in the augmented environment, with 0 representing a low and 6 representing a high sense of presence. Questions were measured along three main themes: realness (Q1-Q2), perceptual stress (Q3-Q4), and spatial presence (Q5-Q6).

**Table 2. Applied scoring system.**

| **PRODUCTION SCALE** | | |
| --- | --- | --- |
| **Movement component** | **Description** | **Error examples for distorted movements** |
| Grip formation | Manipulation knowledge of the object is essential. | Grip incomplete, too narrow, too wide |
| Movement content | Requires successful retrieval of the matching movement and its integration into a movement plan. | Movement produced by wrong body part |
| Movement orientation | Recognition of movement goal and purpose is needed. | Missing distance to the table |
| Spatial orientation | Correct orientation of the movement and the hand in space. | Hand in the wrong plane |
| **INTERACTION SCALE** | | |
| **Description** | Participant actively tries to reach forward, grasp or follow the virtual object | |

Note: Production Scale: The five object representations were analyzed according the presence or absence of four movement components. Each component was rated on a 3-point scale reflecting the degree of accuracy: 0 (incorrect), 1 (distorted), 2 (correct), resulting in a maximum score of 24 points per object and condition after three trials. For each object, distinct criteria were defined which the rater used to determine whether performance on each of the four movement parts should be rated as 0, 1, or 2. Interaction Scale: The presence or absence of interaction was rated on a 2-point scale (0 = no interaction, 1 = interaction), resulting in a maximum score of 3 points per object and condition after three trials.

**Table 3. DILA-S results: Number of patients affected in the different tests of the DILA-S apraxia assessment battery**

| **DILA-S Task** | **Evaluation** |  |  |  |
| --- | --- | --- | --- | --- |
|  | no | mild | moderate | severe |
| Imitation Meaningless | 1 | 5 | 3 | 12 |
| Imitation Meaningful | 7 | 2 | 4 | 8 |
| Pantomime  Production  Execution | 5  6 | 2  0 | 6  6 | 8  9 |
| Novel Tools  Selection  Production  Execution |  |  |  |  |
|  | 10 | 8 | 1 | 2 |
|  | 15 | 3 | 0 | 3 |
|  | 15 | 2 | 1 | 3 |
| Familiar Tools  Selection  Production  Execution |  |  |  |  |
|  | 15 | 2 | 3 | 1 |
|  | 7 | 0 | 3 | 11 |
|  | 8 | 2 | 5 | 6 |
| NAT Breakfast Task | 8 | 3 | 2 | 7 |

**Table 4. Spearman correlations (r_s_) between Production and Interaction scales.**

| **Screen Environment** | | | | | | | | |
| --- | --- | --- | --- | --- | --- | --- | --- | --- |
|  | Production Screen^Stat^ | | Interaction Screen^Stat^ | | Production Screen^Dyn^ | | Interaction Screen^Dyn^ | |
|  | r_s_ | *p* | r_s_ | *p* | r_s_ | *p* | r_s_ | *p* |
| Production Screen^Stat^ |  |  | -.098 | .672 |  |  |  |  |
| Interaction Screen^Stat^ | --- | --- |  |  |  |  |  |  |
| Production Screen^Dyn^ |  |  |  |  |  |  | .699^*^ | <.001 |
| Interaction Screen^Dyn^ |  |  |  |  | -.10 | .618 |  |  |

| **HMD Environment** | | | | | | | | |
| --- | --- | --- | --- | --- | --- | --- | --- | --- |
|  | Production HMD^Stat^ | | Interaction HMD^Stat^ | | Production HMD^Dyn^ | | Interaction HMD^Dyn^ | |
|  | r_s_ | *p* | r_s_ | *p* | r_s_ | *p* | r_s_ | *p* |
| Production HMD^Stat^ |  |  | .228 | .321 |  |  |  |  |
| Interaction HMD^Stat^ | -.537** | .008 |  |  |  |  |  |  |
| Production HMD^Dyn^ |  |  |  |  |  |  | .278 | .222 |
| Interaction HMD^Dyn^ |  |  |  |  | -.248 | .254 |  |  |

Note: White cells display patients´ values (N=21); Grey marked cells display healthy controls´ values (N=23).

In the control group, the Screen^Stat^ correlation analysis is not displayed because outliers resulted in a false negative correlation (rs=-.429, p=.041). After the exclusion of three extreme outliers, the Interaction scale variable became constant.

*. Correlation is significant at the 0.05 level (2-tailed). **. Correlation is significant at the 0.01 level (2-tailed).

**Table 5. Correlations between Pantomime Effects and patient’s clinical data.**

|  | MMSE (N=16) | | MI (N=21) | | NHPT (N=20) | | Circles test (N=18) | | Stroke onset (N=21) | |
| --- | --- | --- | --- | --- | --- | --- | --- | --- | --- | --- |
|  | r | Sig. | r_s_ | Sig. | r_s_ | Sig. | r_s_ | Sig. | r | Sig. |
| DYN Effect Production | -.140 | .605 | -.144 | .532 | -.695 | <.001** | .524 | .026 | .336 | .137 |
| HMD Effect Production | -.107 | .694 | -.036 | .877 | -.1.91 | .420 | .180 | .474 | -.207 | .368 |
| HOLO Effect Production | -.242 | .366 | -.174 | .450 | -.420 | .066 | .396 | .103 | -.099 | .741 |
| DYN Effect Interaction | .550 | .027* | .257 | .260 | -.014 | .952 | .095 | .709 | .097 | .676 |
| HMD Effect Interaction | .058 | .831 | -.206 | .369 | .039 | .870 | .410 | .091 | -.029 | .902 |
| HOLO Effect Interaction | .236 | .379 | -.243 | .290 | -.011 | .965 | .449 | .061 | -.032 | .891 |

Abbreviations: MMSE: Mini Mental State Examination, MI: Motricity Index, NHPT: Nine Hole Peg Test (left hand), r= Pearson correlation, r_s_ = Spearman correlation

Note: Grey cells display correlations with Production scores. White cells display correlations with Interaction scores. P13 has been removed from the NHPT correlation analysis because of being an extreme outlier. When keeping the outlier, the effect becomes smaller and less significant (r_s_=-.486, p=.026).

**. Correlation is significant at the 0.01 level (2-tailed). *. Correlation is significant at the 0.05 level (2-tailed).

**Table 6. Spearman correlations with Pantomime Effects and DILA-S scores.**

|  | DYN Effect  Production | | HMD Effect  Production | | HOLO Effect  Production | | DYN Effect  Interaction | | HMD Effect  Interaction | | HOLO Effect  Interaction | |
| --- | --- | --- | --- | --- | --- | --- | --- | --- | --- | --- | --- | --- |
|  | r_s_ | Sig. | r_s_ | Sig. | r_s_ | Sig. | r_s_ | Sig. | r_s_ | Sig. | r_s_ | Sig. |
| Imitation Meaningless (N=21) | .324 | .152 | .156 | .500 | .211 | .360 | .185 | .422 | .161 | .485 | .121 | .603 |
| Imitation Meaningful (N=21) | .267 | .242 | -.050 | .828 | .083 | .720 | .153 | .509 | -.068 | .771 | -.164 | .476 |
| Pantomime Production (N=21) | -.098 | .672 | -.066 | .777 | -.082 | .725 | .378 | .091 | -.059 | .798 | -.155 | .501 |
| Pantomime Execution (N=21) | -.148 | .521 | -.039 | .868 | -.052 | .824 | .360 | .109 | -.057 | .809 | -.158 | .493 |
| NTT Selection (N=21) | -.498* | .021 | -.298 | .189 | -.400 | .073 | .073 | .752 | .024 | .918 | -.196 | .395 |
| NTT Production (N=21) | -.066 | .777 | .190 | .408 | -.075 | .748 | .288 | .205 | .030 | .899 | -.023 | .922 |
| NTT Execution (N=21) | .098 | .672 | .162 | .484 | -.059 | .799 | .363 | .105 | -.034 | .884 | -.012 | .959 |
| FTT Selection (N=21) | .067 | .772 | -.163 | .480 | -.269 | .238 | .510* | .018 | .215 | .350 | .233 | .309 |
| FTT Production (N=21) | -.123 | .595 | -.347 | .123 | -.395 | .077 | .076 | .744 | -.117 | .612 | -.181 | .431 |
| FTT Execution (N=21) | -.250 | .274 | -.361 | .108 | -.371 | .098 | -.021 | .928 | -.170 | .460 | -.311 | .170 |
| NAT (N=20) | -.129 | .589 | -.146 | .539 | -.196 | .407 | .546* | .013 | .135 | .572 | 131 | .581 |

Abbreviations: FTT: Functional Tools Test, NTT: Novel Tools Test, NAT: Naturalistic Action Task, r_s_ = Spearman correlation

*. Correlation is significant at the 0.05 level (2-tailed).

**Table 7. Pearson correlation of sense of Presence and Pantomime scores in patients.**

|  | Presence Screen^Stat^  (N=16) | | Presence Screen^Dyn^  (N=15) | | Presence HMD^Stat^  (N=14) | | Presence HMD^Dyn^  (N=14) | | |
| --- | --- | --- | --- | --- | --- | --- | --- | --- | --- |
|  | r | *p* | r | *p* | r | *p* | r | *p* | |
| Production Screen | -.198 | .462 | -.113 | .690 |  |  |  |  | |
| Interaction Screen | .002 | .995 | .057 | .840 |  |  |  |  | |
| Production HMD |  |  |  |  | .381 | .181 | .534* | .049 | |
| Interaction HMD |  |  |  |  | .062 | .834 | .294 | .308 | |
| Note: For correlation analysis in patients, 14 complete data sets were available for HMD environments, 15 for Screen^Dyn^, and 16 for Screen^Stat^.  Abbreviations: HMD = Head Mounted Display, r = Pearson correlation  *. Correlation is significant at the 0.05 level (2-tailed). | | | | | | | | |  |
